# Human Tibroviruses: Commensals or Lethal Pathogens?

**DOI:** 10.3390/v12030252

**Published:** 2020-02-25

**Authors:** Jens H. Kuhn, Hào Pān, Charles Y. Chiu, Matthew Stremlau

**Affiliations:** 1 Integrated Research Facility at Fort Detrick, National Institute of Allergy and Infectious Diseases, National Institutes of Health, Fort Detrick, Frederick, MD 21702, USA; 2 Shanghai Municipal Center for Disease Control and Prevention, Shanghai 200336, China; panhao@scdc.sh.cn; 3 Division of Infectious Diseases, University of California, San Francisco, CA 94143, USA; charles.chiu@ucsf.edu; 4 Equator Labs Incorporated, Washington, DC 20011, USA

**Keywords:** Bas-Congo virus, Beatrice Hill virus, Bivens Arm virus, Coastal Plains virus, Ekpoma virus, rhabdovirus, Sweetwater Branch virus, Tibrogargan virus, tibrovirus, TIBV

## Abstract

Rhabdoviruses are a large and ecologically diverse family of negative-sense RNA viruses (*Mononegavirales*: *Rhabdoviridae*). These viruses are capable of infecting an unexpectedly wide variety of plants, vertebrates, and invertebrates distributed over all human-inhabited continents. However, only a few rhabdoviruses are known to infect humans: a ledantevirus (Le Dantec virus), several lyssaviruses (in particular, rabies virus), and several vesiculoviruses (e.g., Chandipura virus, vesicular stomatitis Indiana virus). Recently, several novel rhabdoviruses have been discovered in the blood of both healthy and severely ill individuals living in Central and Western Africa. These viruses—Bas-Congo virus, Ekpoma virus 1, and Ekpoma virus 2—are members of the little-understood rhabdoviral genus *Tibrovirus*. Other than the basic genomic architecture, tibroviruses bear little resemblance to well-studied rhabdoviruses such as rabies virus and vesicular stomatitis Indiana virus. These three human tibroviruses are quite divergent from each other, and each of them clusters closely with tibroviruses currently known only from biting midges or healthy cattle. Seroprevalence studies suggest that human tibrovirus infections may be common but are almost entirely unrecognized. The pathogenic potential of this diverse group of viruses remains unknown. Although certain tibroviruses may be benign and well-adapted to humans, others could be newly emerging and produce serious disease. Here, we review the current knowledge of tibroviruses and argue that assessing their impact on human health should be an urgent priority.

## 1. Introduction

Rhabdoviriuses, the members of the mononegaviral family *Rhabdoviridae*, form a large and ecologically diverse group of negative-sense RNA viruses that typically produce enveloped virions. They are capable of infecting complex animals (birds, fish, reptiles, mammals) and a wide variety of plants [1,2,3,4]. The family, by far the largest of the order *Mononegavirales*, currently includes 20 genera [5]. Another 10 genera have already been officially proposed [6], and the family is expected to expand further to accommodate dozens of previously identified but yet-unclassified viruses [4]. In addition, high-throughput sequencing has revealed the integration of rhabdovirus-like elements into some arthropods and plant genomes—suggesting an ancient co-evolutionary history of rhabdoviruses with their hosts [7,8,9]. However, the majority of rhabdoviruses are uncharacterized, and many of these viruses are known from (meta)genomic sequencing only.

Although collectively rhabdoviruses have a broad host range, only a few are known to infect humans. Perhaps the best-known human rhabdovirus is rabies virus (RABV; genus *Lyssavirus*), which causes rabies, an acute encephalitis. About 25,000–159,000 people per year die from this disease, and the estimated annual economic cost is $8.6 billion USD [10,11]. Several other lyssaviruses (i.e., Australian bat lyssavirus, Duvenhage virus, European bat lyssaviruses 1 and 2, Irkut virus, Mokola virus) are known to cause severe rabies-like or less severe febrile diseases in humans, but the incidence of infection overall is very low [12,13,14,15,16].

Chandipura virus (CHPV; genus *Vesiculovirus*) has caused several outbreaks of fatal encephalitis among humans in India [17,18]. These outbreaks have affected hundreds of people with case fatality rates of approximately 50% [19]. Vesicular stomatitis Indiana virus (VSIV; genus *Vesiculovirus*) is an important livestock pathogen and generally produces mild influenza-like clinical signs in humans [20]. However, one case of human encephalitis due to VSIV infection has been documented [21]. Human exposure to VSIV is generally assumed to be rare, but several studies indicate that exposure to VSIV, as well as other vesiculoviruses, is actually very high in certain areas. In Panama, VSIV-neutralizing antibodies rates in humans were reported to range from 3% in Chupampa, Herrera Province, to 94% in El Aguacate, Chiriquí Province [22,23,24]. In Iran, serological studies of Isfahan virus (ISFV) found that exposure rates among various communities varied from 0–86% [25,26]. In Columbia, vesicular stomatitis Alagoas virus (VSAV)-neutralizing antibody rates for two surveyed towns were 63% and 83%, respectively [27]. Finally, in Brazil, Piry virus (PIRYV) seroprevalence ranged from 3% in Rondônia State to 40% in Rio Grande do Sol State [28,29,30].

Next to lyssaviruses and vesiculoviruses, viruses of a third rhabdoviral genus, *Ledantevirus*, have also been associated with human infections. Le Dantec virus (LDV) was isolated in 1965 at Le Dantec University Hospital in Dakar, Senegal, from serum of a 10-year-old girl with fever and hepatosplenomegaly [31]. A second infection appeared in 1969 in a 47-year-old dock worker in South Wales, United Kingdom, after he had been bitten in the abdomen by an insect. The patient experienced tremors in the right hand and was treated for Parkinson’s disease. A battery of antibody tests were performed and all were negative—except for antibodies that specifically reacted with LDV [32]. Epidemiological studies of vesiculoviruses and ledanteviruses across multiple regions of the world have not been rigorously pursued, and the impact of these viruses on human health may be underappreciated.

Since 2012, RNA genomes of three novel rhabdoviruses, called Bas-Congo virus (BASV), Ekpoma virus 1 (EKV-1), and Ekpoma virus 2 (EKV-2), have been discovered by metagenomic next-generation sequencing (mNGS) of plasma samples from humans in Central or Western Africa [33,34]. BASV was found in a severely ill male in the Democratic Republic of the Congo and EKV-1/2 were found in two healthy females in Nigeria. All three viruses cluster phylogenetically with poorly characterized rhabdoviruses of the genus *Tibrovirus*. Eight genetically distinct tibroviruses are known, including the three viruses associated with humans [33,34,35,36,37,38]. All tibroviruses share a similar genome organization containing at least three unique, genus-specific open reading frames (ORFs) of unknown function [3,39,40,41,42]. The majority of nonhuman tibroviruses have been isolated from biting midges (Diptera: Ceratopogonidae: *Culicoides*), although one tibrovirus has been isolated from a healthy steer (Artiodactyla: Bovidae: *Bos taurus* Linnaeus, 1758). Seroprevalence studies suggest that infection of cattle is likely to be very common [35,36,37,39,43,44,45,46]. Until the discovery of BASV and EKV-1/2, tibroviruses have been largely ignored because none of them appeared to produce overt disease in cattle or to be capable of infecting humans.

## 2. Discovery of the First Tibroviruses

Tibrogargan virus (TIBV), the first tibrovirus discovered, was isolated from a pool of biting midges (*Culicoides brevitarsis* Kieffer, 1917) that often feed on cattle. The biting midges were collected in 1976 close to Mount Tibrogargan in Peachester, Queensland, Australia [36]. The virus appears to be widespread in Australia. A survey of more than 3000 serum samples from Australian cattle using a virus neutralization test revealed many herds were 100% seropositive [35,36]. Anti-TIBV antibodies were also detected in buffaloes and a Floridian white-tailed deer; but they were not detected in humans, camels, goats, horses, sheep, pigs, wallabies, possums, or dogs [36,37,43,44].

Although exposure to TIBV is common among cattle, no association with disease has ever been found [36]. Cattle herds were tested for seroconversion to TIBV, and although up to 80% of some herds seroconverted, no illness was observed [36]. In addition, sera from calves with arthrogryposis and hydranencephaly and steers with unexplained fevers were tested for the presence of anti-TIBV antibodies. However, no antibodies were found [36]. Experimental infections of cattle produced subclinical viremia without overt signs of illness [35,39,43,44].

In 1981, a second tibrovirus was discovered at the Coastal Plains Research Station (the Coastal Plains Research Farm today) in Northern Territory, Australia [35,45]. This new virus, called Coastal Plains virus (CPV), was isolated from the blood of a healthy, asymptomatic steer more than 3000 km from where TIBV was discovered. CPV-neutralizing antibodies were detected in sera from healthy cattle throughout Australia and Papua New Guinea, suggesting a similar geographical range as TIBV [35,45]. Anti-CPV antibodies were also found in water buffaloes, dogs, and one horse, but not in deer, pigs, or wallabies [35]. Like TIBV, no antibodies were detected in humans, and no disease was associated with CPV in any animal tested [35,45].

From 1981–1982, two tibroviruses, Sweetwater Branch virus (SWBV) and Bivens Arm virus (BAV), were isolated from pools of biting midges (*Culicoides insignis* Lutz, 1913) feeding on water buffaloes recently imported into Florida from Trinidad [37]. Approximately 40% of serum samples from the water buffaloes contained antibodies capable of neutralizing BAV and/or TIBV [37,43]. Cattle in Florida also tested positive for neutralizing antibodies to BAV (≈23% positive) and TIBV (≈22% positive) [37,43]. Anti-BAV antibodies were also detected in healthy cattle from Puerto Rico and the Virgin Islands, USA, and in one horse and one white-tailed deer in the US [46]. The discovery of BAV and SWBV in Floridian biting midges and evidence of neutralizing antibodies in animals throughout Florida and the Caribbean suggest that tibroviruses may be globally distributed. However, no evidence of clinical disease in water buffaloes, sheep, wildebeest, or cattle was associated with SWBV or BAV [46].

In 1984, a fifth tibrovirus was reported. This virus, Beatrice Hill virus (BHV), was isolated from a pool of biting midges (*Culicoides peregrinus* Kieffer, 1910) originally collected at Beatrice Hill, Northern Territory, Australia from 1974–1976 [38]. Serological studies have not been conducted to assess the exposure of cattle and other animals to BHV.

### 2.1. Bas-Congo Virus

**Figure 1:**
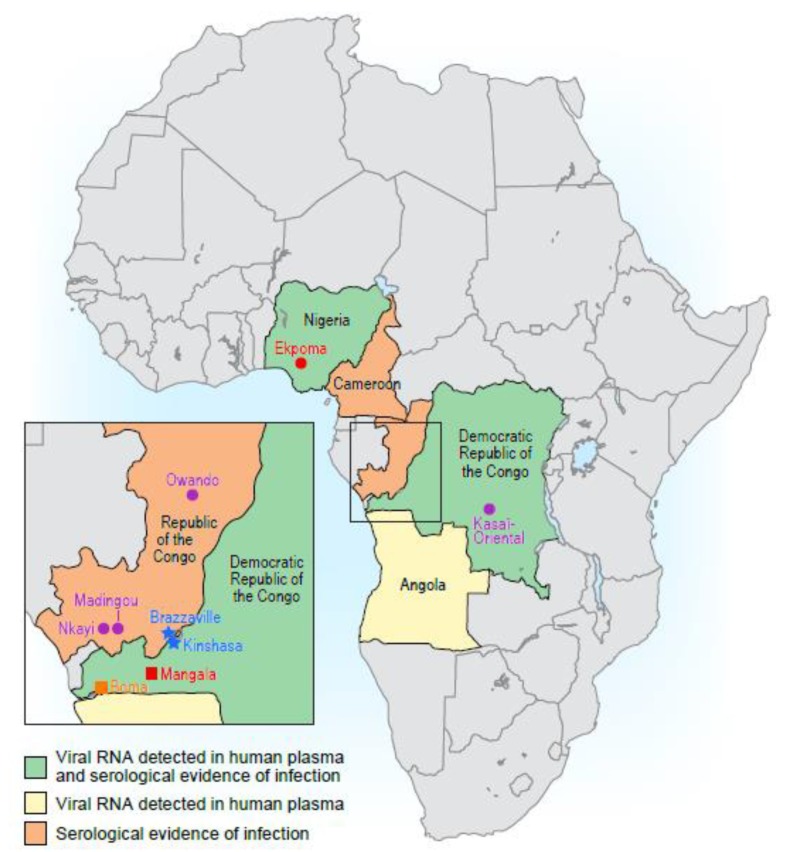
Human-associated tibroviruses in Africa. Map of Africa showing countries where tibrovirus RNA has been detected in human blood samples and/or where serological evidence of tibrovirus infections exists. Mangala village, the site of the 2009 cluster of cases of unexplained fevers that led to the discovery of BASV, is represented by a red box. Boma, the city where the blood sample that contained BASV RNA was collected, is indicated by an orange box. Ekpoma, the town where blood samples containing EKV-1 and EKV-2 RNA were collected is represented by a red circle. Sites of BASV serosurveys are represented by purple circles. The capitals of the Democratic Republic of the Congo and the Republic of the Congo are denoted by blue stars.

On 25 May 2009, a 15-year-old boy in Mangala, Bas-Congo (today Kongo Central) Province, Democratic Republic of the Congo (Figure 1) developed symptoms and clinical signs resembling acute viral hemorrhagic fever [33,47,48]. The patient’s presentation included malaise, gingival and nasal bleeding, hematemesis, conjunctival injection, and bloody diarrhea. The patient died 2 days after the onset of symptoms.

Eleven days later, on 5 June 2009, a second patient, a 13-year-old girl, arrived at the same health clinic with headache, fever (>39 °C), abdominal pain, gingival and nasal bleeding, hematemesis, conjunctival injection, and bloody diarrhea [33,47,48]. She had attended the same public school as the first patient, but contact between the two of them could not be substantiated. This patient died within 3 days after disease onset.

More than a week later, on 13 June 2009, a 32-year-old male nurse who cared for the two teenagers fell ill [33,47,48]. His clinical presentation also resembled viral hemorrhagic fever and included nasal, ocular, and oral bleeding; hematemesis; and bloody diarrhea. He was immediately transported to an intensive care unit in the nearby city of Boma and was given supportive treatment, including antibiotic(s). Remarkably, he recovered and became the lone survivor from this small cluster of clinically similar cases. RNA was extracted from an acute serum sample collected from the nurse upon his admittance and subjected to mNGS. Assembly of the viral reads revealed the presence of a novel rhabdovirus, BASV, that was phylogenetically similar to tibroviruses [33]. The BASV plasma titerwas 1.09 × 10^6^ RNA copies/mL based on an *L* gene -specific reverse transcription-quantitative polymerase chain reaction (RT-qPCR) assay [33]. This viral titer was modest and may reflect the survival status of this patient. A virion-neutralization test using VSIV particles pseudotyped with the BASV envelope glycoprotein (G) indicated the presence of neutralizing antibodies in the nurse’s plasma and in one of five asymptomatic health care workers who had been in close contact with him [33]. The presence of neutralizing antibodies suggests previous virus exposure but does not indicate the presence or absence of a prior associated illness. No samples were available from the 13-year-old boy or the 15-year-old girl. Thus, although all three individuals lived within 50 m of each other in Mangala, a direct connection between these three cases and BASV infection of the first two cases could not be established.

Despite attempts to isolate BASV by inoculating cultures of Asian tiger mosquito (*Aedes albopictus* Skuse, 1894) mosquito larva (C6/36), European rabbit (*Oryctolagus cuniculus* Linnaeus, 1758) kidney epithelial (LLC-RK1), grivet (*Chlorocebus aethiops* Linnaeus, 1758) kidney epithelial (Vero), golden hamster (*Mesocricetus auratus* Waterhouse, 1839) kidney fibroblast (BHK-21), and rhesus monkey (*Macaca mulatta* Zimmermann, 1780) kidney epithelial (LLC-MK2) cells with BASV-positive sera from the nurse, none of the cultures developed overt cytopathic effects (CPE). Evidence of BASV replication could not be detected by RT-qPCR [33]. Furthermore, no evidence of illness or deaths occurred in suckling laboratory mice inoculated intracerebrally with the BASV-positive serum [33]. Explanations for these negative results include the possibility that samples may have experienced fluctuating temperatures that may have compromised viral particles in the samples or the cell lines, and that mouse models typically used for arbovirus isolation do not support BASV replication. Unfortunately, further isolation attempts cannot be performed because all available, non-inactivated clinical material was depleted.

Metagenomic sequencing is a powerful approach for identifying viruses, including unknown ones, but detection alone may not unambiguously establish the cause of a patient’s illness [49]. In the case of BASV, only plasma was surveyed, but other tissues may also harbor the disease-causing pathogen. Furthermore, Médecins Sans Frontières reported 121 cases of hemorrhagic diarrhea with fever in the area surrounding Mangala Village around May—June 2009. Shigellae were detected in two of these cases (Lampaert Emmanuel to Robert Garry, personal communication) [50], suggesting that other plausible causes are possible for the unexplained illnesses observed in these three human cases of BASV infection. Further epidemiological studies, ideally resulting in re-discovery and possibly isolation of BASV or creation of replicative BASV in the laboratory, are needed to establish the link between this virus and human disease.

### 2.2. Ekpoma Viruses 1 and 2

In 2015, the discovery of two more novel human-associated rhabdovirids was reported [34]. These viruses, EKV-1 and EKV-2, were identified in plasma samples from two healthy females in Ekpoma, Edo State, Nigeria. This finding was not anticipated, as detection of RNA viruses, unlike DNA viruses, is rare in the plasma of healthy people [51,52,53,54]. RNA viruses present in such plasma are usually only those viruses that cause chronic infections, such as retroviruses, pegiviruses (*Flaviviridae*), or hepaciviruses (*Flaviviridae*) [55]. Even more concerning was that two individuals harboring novel rhabdoviruses were identified among just 328 samples [34].

EKV-1 and EKV-2 were identified in two females 45-years and 19-years of age, respectively. In neither sample did mNGS analysis reveal the existence of any other RNA virus. At the time of sample collection, neither woman had any indication of illness. Two years after the initial blood draw, oral interviews were conducted with the two women. The woman infected with EKV-1 could not recall any episode of illness in the weeks or months following the initial collection of blood. The woman with EKV-2 recalled suffering an episode of febrile illness 2 weeks after blood collection. At that time, she was admitted to a hospital, and her illness was clinically diagnosed as malaria. Follow-up RT-qPCR for EKV-1 and EKV-2 infection 2 years after the initial blood draws were negative. However, an enzyme-linked immunosorbent assay indicated that both women had antibodies reacting with the EKV-1 and EKV-2 nucleocapsid (N) proteins [34].

Similar to viremia observed with BASV infection, plasma titers observed in both healthy females were modest. EKV-1 and EKV-2 titers were 4.5 × 10^6^ RNA copies/mL and 4.6 × 10^4^ RNA copies/mL, respectively [34]. Infectious viruses could not be isolated from the plasma samples by inoculation of Vero E6, BHK, C6/36 mosquito, LLC-MK2, SW13, and biting midge (*Culicoides variipennis* Coquillett, 1901) cell lines nor by intracranial inoculation of newborn laboratory mice [34]. Similar to BASV, further isolation attempts were impossible because all available, non-inactivated clinical material was depleted.

More recently, an individual in Angola was discovered to be infected with EKV-2 [56]. On 25 December 2016, a male Chinese laborer in Angola presented at a local medical clinic with chills, mild pain in the calf joints, and a 39 °C fever. He was suspected of having yellow fever and was sent to China for further treatment. Upon arriving in China on 2 January 2017, his blood was tested for dengue and yellow fever viruses (*Flaviviridae*: *Flavivirus*), hepatitis A virus (*Picornaviridae*: *Hepatovirus*), hepatitis B virus (*Hepadnaviridae*: *Orthohepadnavirus*), hepatitis C virus (*Flaviviridae*: *Hepacivirus*) Rift Valley fever virus (*Phenuiviridae*: *Phlebovirus*), chikungunya virus (*Togaviridae*: *Alphavirus*), and plasmodium infection. All tests were negative [56]. mNGS analysis of RNA extracted from the man’s blood revealed several sequence contigs with homology to EKV-2 [56]. Fifteen days later, on 17 January 2016, another blood sample was subjected to mNGS analysis. Deeper sequencing revealed a coding-complete genome sequence that was 96.4% identical to EKV-2 [56]. The individual eventually recovered. No additional RNA viruses were reported in the patient’s plasma samples, and no information was made available about the patient’s viral load. Whether EKV-2 virus caused the patient’s illness remains undetermined. Variation in the viral genome, host genetics, and lack of prior exposure to EKV-2 could be potential reasons why EKV-2 could be associated with illness in the Chinese patient and not the Nigerian EKV-2-infected woman.

## 3. Exposure to Human Tibroviruses

Human-associated tibroviruses may be common and potentially widespread throughout Western and Central Africa (Figure 1, Table 1). A serosurvey using an enzyme-linked immunosorbent assay designed to detect antibodies that recognize the N protein of EKV-1 and EKV-2 suggested that 5% of people living in and around Ekpoma had been exposed to EKV-1 and 45% had been exposed to EKV-2 [34]. EKV-1 and EKV-2 antibodies were detected in less than 1% of plasma samples collected from people living in the US, suggesting very little or no exposure to these viruses. Limited cross-reactivity was observed between assays for EKV-1 and EKV-2. However, strong cross reactivity was observed between assays for EKV-1 and RABV [34]. This result suggests that other rhabdoviruses or undiscovered rhabdoviruses circulating in Nigeria could potentially inflate EKV-1 and EKV-2 seroprevalence.

To date, EKV-2 RNA has been detected in plasma samples from three individuals: the woman in Nigeria [34], the Chinese worker in Angola [56], and one healthy nurse in the Democratic Republic of the Congo (Table 1). The detection of viral RNA in these countries suggests that EKV-2 is circulating within a vast geographic area that includes more than 300 million people.

Human exposure to BASV appears to be geographically limited. A serosurvey of 50 randomly-selected blood donors from the Kasaï-Oriental Province in Democratic Republic of the Congo did not detect any positive samples [33]. This province, however, is located ≈2,000 km from the town were BASV was identified. A different serosurvey in the Republic of the Congo found 18/458 human plasma samples contained antibodies capable of neutralizing a VSIV virion pseudotyped with BASV G, indicating a seroprevalence rate of 4% (Graham Simmons and Imke Steffen, personal communication). Interestingly, positive samples were only collected from two villages: Nkayi and Madingou in Bouenza Department. Samples from a third village, Owando in Cuvette Department, were all negative [57]. However, cross-reactivity in this study may be significant. For instance, approximately 70% of the positive samples had neutralizing activity against kotonkan virus (KOTV; genus *Ephemerovirus*), an arthropod-borne rhabdovirus isolated from biting midges (*Culicoides* sp.) collected in Ibadan, Oya State, Nigeria, in 1967 [57].

### Transmission of Human Tibroviruses

The natural reservoirs and routes of transmission of BASV and EKV-1/2 are unknown. Because the nonhuman-associated tibroviruses are transmitted by biting midges, an assumption that BASV and EKV-1/2 are similarly transmitted is reasonable. Consequently, human infections may occur via bites. The BASV genome has been recently analyzed with a novel machine learning algorithm designed to predict animal reservoirs and arthropod vectors directly from viral genome sequences [58]. This analysis suggests that the natural host of BASV is an artiodactyl (e.g., cattle) and that BASV may be vectored by biting midges. As biting midges and cattle can be found throughout a region that stretches from Nigeria to Angola, human infection could plausibly be accidental. These infections could be due to spillover from the biting midge-cattle transmission cycle or biting midge infection of some other wild artiodactyl.

Humans could also possibly serve as the natural host reservoir for certain tibroviruses. Humans and arthropod vectors have a long co-evolutionary history in Central and Western Africa. Over many millennia, certain tibroviruses may have adapted to humans. Such a relationship between arboviruses and humans would be unusual but not without precedent. For instance, all four dengue viruses use humans as their primary host reservoir [59]. Humans amplify the viruses and facilitate their transmission to uninfected individuals via mosquito (predominantly yellow fever mosquito—*Aedes aegypti* Linnaeus, 1762) vectors. Perhaps the transmission cycles of well-adapted human-associated tibroviruses are similar.

## 4. Tibrovirus Genome

**Figure 2:**
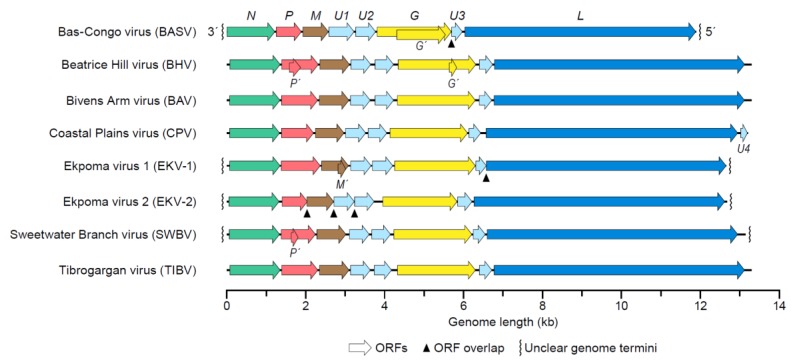
Tibrovirus genome organization. Tibrovirus genomes (drawn to scale) contain the following open reading frames (ORFs): nucleoprotein (*N*), phosphoprotein (*P*), matrix protein (*M*), unknown proteins 1 (*U1–3*), and large protein (*L*). Alternative and overlapping ORFs are at least ≥180 bp (≥60aa) and are transcribed in the same polarity, but in a different frame, within a longer ORF. The 3′-leader (Le) and 5′-trailer (Tr), and additional ORF *U4* of CPV, are also depicted.

Typical rhabdovirions contain a single molecule of linear, negative-sense RNA. The length of the genome is approximately 10–16 kb and encodes at least five proteins: nucleoprotein (N), phosphoprotein (P), matrix protein (M), glycoprotein (G), and large protein (L). The order of the ORFs encoding these proteins (3′-*N-P-M-G-L*-5′) is highly conserved, and each ORF is flanked by relatively conserved transcription initiation and termination sequences [4]. Next to the canonical rhabdoviral ORFs, tibrovirus genomes also contain three tibrovirus-specific ORFs designated U1, U2, and U3 (the CPV genome contains a fourth ORF, U4) [39,40,60]. The function of these accessory genes is unknown [42,61]. Two of the ORFs, U1 and U2, are located between those encoding M and G. The U3 ORF is situated between those encoding G and L (Figure 2). U3 may encode a viroporin, a small hydrophobic protein that oligomerizes to form hydrophilic pores in the host cell membrane [61]. The U1 and U2 proteins may modulate the host response or are involved in transmission, rather than play a role in the structure or replication of the virion. These proteins could be similar to the C and V accessory proteins of measles virus, which are involved in counteracting the host’s antiviral response [62]. The U1–U3 accessory proteins are among the most divergent proteins encoded by BASV and EKV (Table 2), suggesting that they may have evolved to play highly host-specific roles. In addition, four tibroviruses appear to encode additional small proteins from ORFs overlapping with major ORFs; however, the function of these proteins has not yet been analyzed.

The termini of the rhabdovirus genome contain partially complementary, untranslated leader and trailer sequences. The length of the 3′ leader sequence ranges from 67–72 nt, and the 5′ trailer ranges from 168‒250 nt (Table 3). Unfortunately, the genome sequences of BASV, EKV-1, and EKV-2 are incomplete.

### Genetic Variation of Tibroviruses

**Figure 3:**
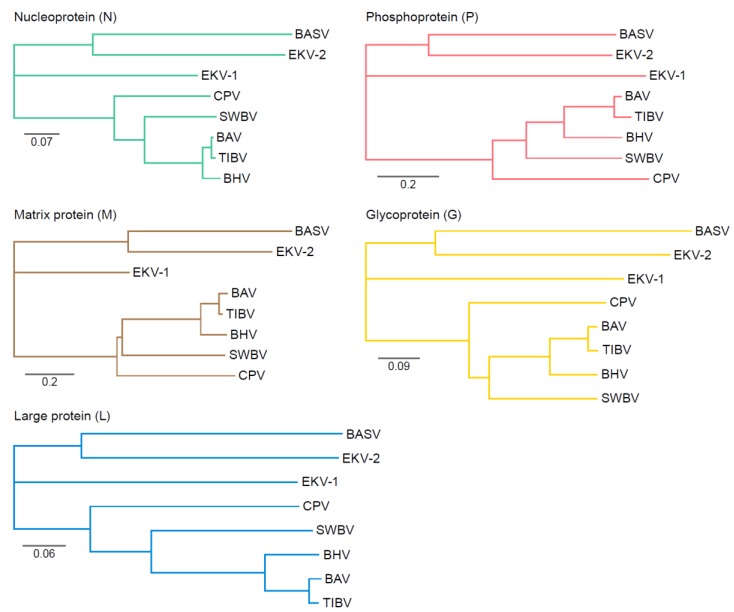
Tibrovirus phylogeny. Multiple sequence alignments of tibrovirus nucleoprotein (N), phosphoprotein (P), matrix protein (M), glycoprotein (G), and large protein (L) full-length amino acid sequences were performed using Multiple Alignment with Fast Fourier Transform (MAFFT). Nearest-neighbor and unrooted trees were generated using Geneious Prime 2019. Scale bar = substitutions/site.

Tibroviruses are genetically divergent [5,33,34,39,40,60], and overall amino acid homology among concatenated ORFs of the human-associated tibroviruses ranges from 33–39% [34]. BASV, BHV, CPV, EKV-1, EKV-2, and SWBV represent unique species (*Bas-Congo tibrovirus*, *Beatrice Hill tibrovirus*, *Coastal Plains tibrovirus*, *Ekpoma 1 tibrovirus*, *Ekpoma 2 tibrovirus*, and *Sweetwater Branch tibrovirus*, respectively); because of their close genetic relationship, BAV and TIBV are considered unique viruses of the same species (*Tibrogargan tibrovirus*) [4]. EKV-2 is slightly more similar to BASV at the amino acid level than to EKV-1. Phylogenetically, EKV-1 appears to be a closer evolutionary relative to the nonhuman associated tibroviruses than to EKV-2 or BASV (Figure 3). EKV-2 and BASV may have undergone more interactions with humans (or some other shared host), whereas EKV-1 may have emerged more recently from a reservoir shared with other tibroviruses. Due to the extreme level of divergence and limited number of tibrovirus genomes, drawing any firm conclusions is not possible. However, this wide diversity also implies that additional tibroviruses remain to be discovered that will occupy branches between the major tibroviral clades thus far described.

The envelope Gs are notably divergent at the amino acid level (Table 2). Although EKV-1 and EKV-2 were discovered in humans of the same community, the envelope Gs are only 26% identical at the amino acid level. By comparison, two of the more divergent vesiculoviruses, ISFV and VSIV, have envelope G that are 49% identical [63].

Phosphoprotein (P) divergence is also notable among tibroviruses (Table 3). Whereas the length of most tibrovirus proteins are similar from virus to virus, and BASV and EKV-2 Ps are ≈33% shorter than those of other tibroviruses. The functional consequences of this shorter length are unclear. P, in addition to playing a highly conserved role in synthesizing viral RNA, also plays a key role in RABV immune evasion by inhibiting different steps of the interferon pathway [64]. Whether this role is also exerted by tibrovirus P remains to be determined.

## 5. Tibrovirion Morphology

**Figure 4:**
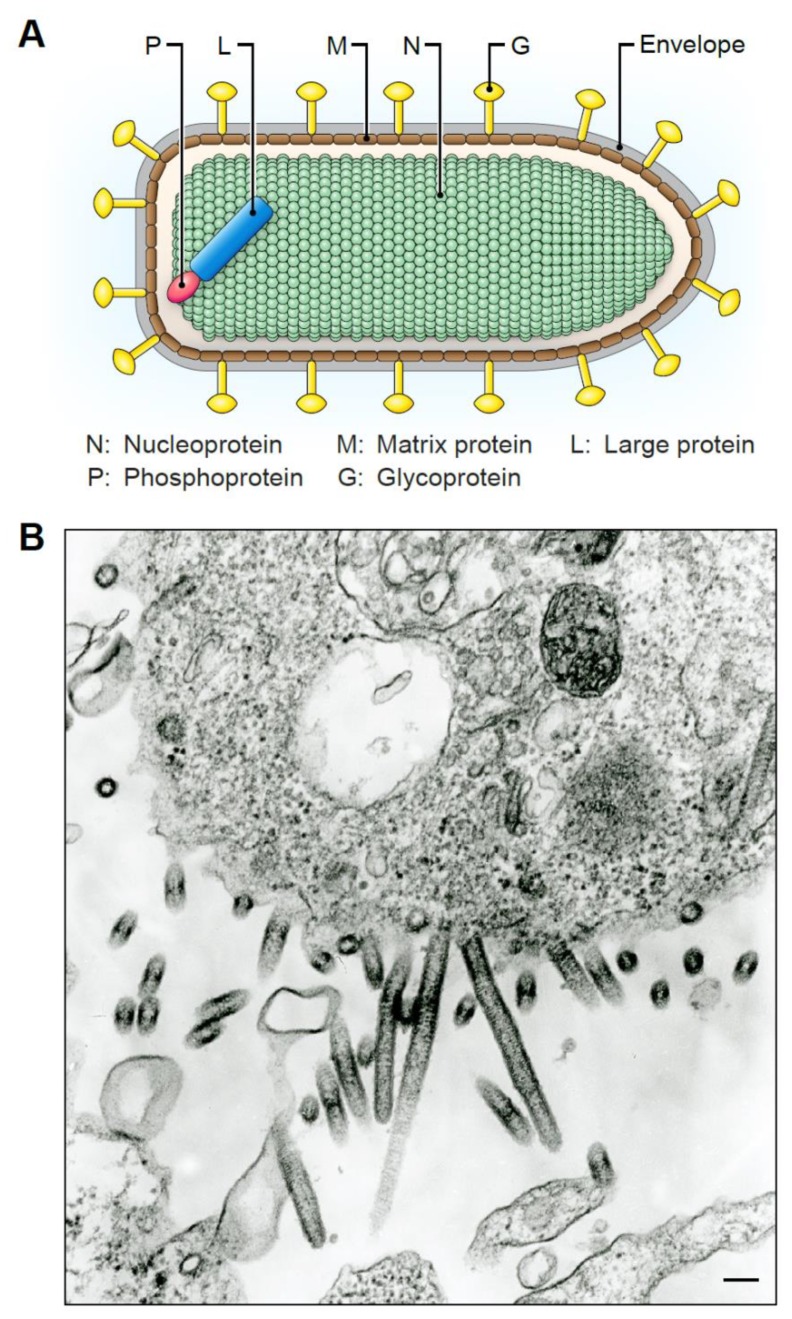
Tibrovirion morphology. (**A**) Illustration of the typical rhabdovirion “bullet.” (**B**) Electron micrograph of Sweetwater Branch virions budding from C6/36 mosquito cells. Magnification × 96,000. Image provided by Vsevolod Popov, University of Texas Medical Branch. Scale bar = 100 nm

Rhabdovirions capable of infecting humans are typically bullet-shaped (Figure 4A), approximately ≈180 nm long, and ≈75 nm wide [4,65,66]. Plant rhabdoviruses, in contrast, often produce particles that appear bacilliform and reach lengths up to 460 nm [67]. Thus, the morphology of both vertebrate and plant rhabdovirions can vary significantly, and some animal-infecting viruses produce particles that can also appear bacilliform.

The virion morphologies of BASV or EKV-1/2 are unknown because these viruses have not been isolated in cell culture, and electron-microscopic (EM) studies could not be performed. However, EM images are available for SWBV and TIBV particles [36] (Figure 4B). SWBV produces very long viral particles in C6/C36 cells. TIBV also forms elongated particles ranging in lengths from 125–375 nm. Electron micrographs are not available for CPV particles; however, the publication describing the isolation of CPV states that “elongated particles were observed” and “a few, very distorted, short, bullet-shaped particles were seen in one sample” [35]. The shapes of these elongated particles are in contrast to the compact bullet shapes of, for instance, RABV and VSIV particles. Tibrovirion morphology should be interpreted with caution as all structures were determined using virions produced in cell culture.

The significance of synthesizing longer, bacilliform particles, if any, is unclear. Synthesis of elongated particles likely requires greater energy, more time, and a host cell less sensitive to the lytic effects of the budding virus. Elongated virions may reflect a well-adapted host-virus relationship, which would be expected for tibroviruses and insect cells. When infectious BASV and EKV-1/2 become available to study, determining whether the production of compact bullet-shapes or elongated bacilliforms is cell type- and/or host-specific will be interesting.

## 6. Tibrovirion Host Cell Entry and Tropism

The mononegaviral envelope G is important because it mediates the entry of viral particles into a cell and establishes infection within the host. The envelope G is also typically the most-exposed virion component and, therefore, the primary target of non-neutralizing and neutralizing antibodies.

The BASV and EKV-1/2 Gs share sequence and structure similarities with Gs of other rhabdoviruses [68]. Their precursors contain an N-terminal signal peptide, a single transmembrane domain, and a short intracellular C-terminus. Tibrovirus Gs are likely class III fusion proteins [68], which are characterized by internal fusion domains and membrane-membrane fusion-induced conformational changes that are fully reversible [69,70,71]. Rhabdovirus Gs are generally not highly glycosylated. RABV and VSIV Gs have only 2–4 *N*-linked glycosylation sites compared to up to 17 on, for instance, the Ebola virus (*Filoviridae*: *Ebolavirus*) glycoprotein [72]. *N*-glycosylation of BASV G (but not other tibroviruses) has been experimentally confirmed and is expressed at a similar low level on Gs of other rhabdoviruses [68].

Rhabdovirus particles are considered to enter cells via pH-dependent, clathrin-mediated endocytosis. Tibrovirion entry, including BASV and EKV, is also pH-dependent [73]. Treatment of target cells with various inhibitors of clathrin-mediated endocytosis inhibited tibrovirus G-mediated cell entry in a dose-dependent manner, suggesting that tibrovirion entry proceeds via a similar mechanism as other rhabdoviruses [68,73]. The overall cell tropism of tibroviruses is likely determined by specific G interactions with host cell virion attachment factors and/or receptors.

Lectins, such as CD209 (DC-SIGN), can serve as attachment factors and enhance infection of certain viruses. Typical rhabdoviruses, such as RABV and VSIV, are not enhanced in the presence of lectins [74]. Unexpectedly, the presence of lectins such as CD209 enhances BASV particle entry nearly 8-fold [68]. This significant enhancement suggests that BASV particles may interact with lectin-expressing cells, such as dendritic cells, by different mechanisms than other rhabdoviruses. Lectin-mediated enhancement of entry of particles of other tibroviruses has not been investigated.

The cellular receptor(s) that mediate tibrovirion entry have not been identified. However, the cellular and tissue tropism of tibrovirion entry has been studied using particles of recombinant G ORF-deficient individual VSIVs that express enhanced green fluorescent protein (rVSIVΔG-eGFP) pseudotyped with or directly expressing each of the tibrovirus Gs [68,73]. These experiments produced unexpected results. VSIV expressing its native G has extremely broad, nearly universal cellular tropism [75,76,77]. rVSIVΔG-eGFP virions expressing each of the tibrovirus Gs also demonstrated remarkably broad tropism in human, nonhuman (e.g., nonhuman primate, bat, rodent, snake), and insect cells (e.g., Asian tiger mosquito cells) [68,73]. The entry efficiency of rVSIVΔG-eGFP particles expressing BASV G were slightly less than that of control rVSIV. However, the entry efficiency of particles harboring EKV-1 or EKV-2 G, however, was dramatically greater than that of the VSIV G control. Even at low multiplicities of infection (MOI)—when infection rates of VSIV expressing its native G sharply decrease—recombinant VISV viruses expressing EKV-1 or EKV-2 G continued to infect cells at high levels [73]. VSIVΔG-GFP particles expressing EKV-1 or EKV-2 were even able to enter several cell lines resistant to VSIV. These results suggest EKV-1 and EKV-2 particles are more efficient at entering human cells than particles of VSIV or any of the other tibroviruses [73].

Although informative, the use of recombinant VSIV particles containing tibrovirus Gs (either single-round or replication competent) should be interpreted carefully. These recombinant viruses can only indicate whether a cell is susceptible to tibrovirus-mediated viral entry, not if a cell is permissive to a full replication cycle. Indeed, data obtained with replication-competent VSIVΔG-GFP expressing various tibrovirus Gs suggest a much wider cell tropism than experiments performed with authentic tibroviruses. This discrepancy indicates that cells of different species have evolved anti-tibrovirus intrinsic immunity (e.g., restriction factor [s]) that inhibit tibrovirus replication after virion cell entry. On the other hand, the results suggest that tibrovirus receptors, like the ones for VSIV, are ubiquitously expressed. Although tibroviruses exhibit broad tropism, differences in entry efficiency are apparent and can be quantified. These differences, along with epidemiological data, may provide insights into the identity of tibrovirus vectors and natural hosts.

## 7. In vitro Tibrovirus Replication and Cytopathic Effects

CPE are (often easily visible) morphological changes in virus-infected cells that may be cell-type dependent. CPE usually refers to morphological changes in two-dimensional cell culture, but many of the changes observed in vitro are likely to occur in vivo and may be relevant to pathogenesis. The extent of CPE varies significantly among rhabdoviruses and by cell type [78]. For instance, VSIV causes extensive CPE on most cell types and is among the most cytopathic mammalian viruses [78]. RABV, in contrast, produces almost no CPE on most cell types [78].

Because infectious, replication-competent isolates of BASV and the EKV-1/2 are unavailable, no information about their ability to replicate in cell culture and produce CPE exists. The only insights into authentic tibrovirus replication come from studies of nonhuman tibroviruses. In 2010, various human cancer cells were exposed to 20 rhabdoviruses from diverse genera to identify oncolytic viruses [79]. TIBV proved to be weakly cytopathic on most cell lines, generally causing observable CPE at MOI 1–10 following 96 h of infection but was highly lytic (CC_50_ < 0.1 MOI) for one neuronal cell line (U373), one ovarian cell line (OVCAR8), and one colon cell line (CT26). VSIV, on the other hand, was nearly 100% lytic at less than 0.1 MOI in under 48 h in most cell lines [79].

A more recent and comprehensive study assessed the replication of infectious BAV, CPV, SWBV, and TIBV on a broad panel of human, nonhuman primate, rodent, snake, and Asian tiger mosquito cells [73]. TIBV produced CPE in most human cell lines, and N expression could be detected with an anti-TIBV antibody in western blots. Notably, TIBV did not produce CPE in most nonhuman primate cell lines, but low concentrations of N could be detected. The other tibroviruses (e.g., BAV, SWBV, and CPV) were unable to replicate or express N and did not produce CPE in most human and nonhuman cell lines even though they can successfully enter the cells [73]. The nature of the block to these viruses in human cells remains to be elucidated.

Tibroviruses have been cultured extensively on insect cells (*Culicoides brevitaris*, C6/36), grivet Vero cells, and laboratory mice during the process of viral isolation and passaging over time [73]. Genetic adaptations to these cells may have influenced their ability to complete a full replication cycle in human and nonhuman cells. Thus, the replication of wild-type tibroviruses in cells from their natural host may produce starkly different results. Low-level replication and weak CPE on human cells may be a defining feature of certain tibroviruses. This feature may explain their ability to infect humans without causing observable clinical signs.

## 8. Implications for Human Health

Except for RABV, rhabdoviruses are not considered a public health priority. Very few rhabdoviruses are known to infect humans, and exposure to these viruses is considered rare. The discovery of several novel rhabdoviruses (BASV, EKV-1/2) in human plasma samples raises the possibility that humans may interact more frequently with rhabdoviruses than is generally recognized. The impact of these infections could span a wide range of clinical outcomes from benign to fatal. Host genetics and environmental factors may play a major role in determining these outcomes. Certain rhabdoviruses may possibly be beneficial to humans by providing protection against more virulent viruses.

Additional tibroviruses with the capability of infecting and replicating in humans almost certainly exist, as suggested by the large phylogenetic distances between human tibroviruses, and the cross-reactivity observed in some serological assays. Some of these viruses—perhaps like EKV-2—could be well-adapted to humans and not produce any discernable clinical signs upon infection. Other tibroviruses—perhaps BASV—could be newly emerging and produce severe disease. Tibroviruses could possibly be opportunistic (i.e., they could only establish viremia when their hosts’ immune systems are compromised). Many arboviruses—such as West Nile virus, dengue viruses, or Zika virus (*Flaviviridae*: *Flavivirus*)—produce extremely mild or no clinical signs in the vast majority of infected individuals [80]. Thus, clinical manifestations, whether mild or severe, are possibly rare even though infections with BASV and EKV may be very common.

Although many of these questions cannot be answered at this time, several features of human-associated tibroviruses infections are worth emphasizing. First, researchers did not expect detection of rhabdovirus RNA in human plasma because rhabdoviruses generally do not produce detectable viremia in mammalian hosts. For instance, RABV viremia does not occur in natural infections, and the presence of a viremic state in humans is controversial [81,82,83]. Vesiculoviruses also do not produce a viremic state in primate hosts [84] or even in experimental infection of cattle [85]. On the other hand, most arbovirus infections of humans produce at least a transient viremia. RNA viruses are typically present in blood for several days or more before clearance by the immune system.

The discovery of EKV-1/2 was even more unexpected considering that the two virus genomes were discovered by screening just 328 samples from apparently healthy individuals. This finding suggests that rhabdovirus infection must be very frequent or that tibrovirions (and thereby their RNA genome) are able to persist for long periods in the blood. Evidence is growing that viral RNA can persist in certain tissues long after acute infection has ended. For instance, in experimental measles virus infection of rhesus monkeys, non-infectious RNA can persist in peripheral blood mononuclear cells (PBMCs) for up to 70 days after cessation of acute viremia [86]. West Nile virus can persist in the kidneys for many months [87]. Most relevant perhaps is that VSIV RNA has been observed to persist in the lymph nodes of infected laboratory mice for at least 6 months [88].

In the Nigerian and Democratic Republic of the Congo cases, tibroviral load was modest (ranging from tens of thousands of viral RNA copies to several million). These RT-qPCR results need to be critically examined because none of these assays are clinically validated, nor do they measure genome copies specifically (instead measuring the polymerase (L) gene product, which captures both the number of genome copies as well as the number of L transcripts). Qualitatively, however, the results suggest that in the tested individuals tibroviral load was indeed modest, perhaps reflecting low-level infection, convalescence of an acute infection, or low levels of persistent non-infectious tibrovirus RNA. Low level replication could be a reason why the 32-year-old nurse survived BASV infection or the reason why neither of the Nigerian women infected with EKV-1/2 showed any signs of illness. It is also possible that low-level replication reflects a commensal state and BASV and EKV-1/2 produce benign infections that have nothing to do with the true cause of illness.

In summary, the impact of tibrovirus infection on human health remains unknown. Further epidemiological studies and replication-competent BASV and EKV-1/2 are needed to assess the importance and impact of these viruses on human health. Many chronic, autoimmune, and neurological diseases still have no known etiology. Widespread and frequent exposure to RNA viruses capable of infecting humans, such as tibroviruses, may be linked to some of these still unsolved syndromes, which is why “exotic” viruses should not be ignored.

## Figures and Tables

**Table 1:** Human-associated tibroviruses detected in plasma samples from different individuals.

Virus	Country	Year	Clinical Status	Sequence (nt)	Genome Status	GenBank
BASV	Democratic Republic of the Congo	2009	Febrile	11,892	Near-complete	JX297815.1
EKV-1	Nigeria	2013	Healthy	13,158	Coding-complete	KP324827.1
EKV-2	Nigeria	2013	Healthy	12,707	Coding-complete	KP324828.1
EKV-2	Democratic Republic of the Congo	2014	Healthy	1228	Partial	KY766247.1–KY766250.1
EKV-2	Angola	2016	Febrile	12,638	Coding-complete	MF079256.1

**Table 2:** Percent pairwise amino acid homology of proteins encoded by human-associated tibroviruses ^1^.

Virus Pair	N	P	M	U1	U2	G	U3	L
**BASV × EKV-1**	41	19	23	13	15	30	13	43
**BASV × EKV-2**	46	30	28	20	17	34	13	49
**EKV1 × EKV-2**	41	17	22	18	18	26	20	43
**EKV-2 (Nigeria) × EKV-2 (Angola)**	99	100	98	99	99	98	90	98

^1^ Values represent percent (%) pairwise amino acid homology. Alignments were performed using Multiple Sequence Comparison by Log-Expectation (MUSCLE 3.8).

**Table 3:** Tibrovirus protein lengths ^1^.

Virus	Leader (nt)	N	P	M	U1	U2	G	U3	L	Trailer (nt)
**EKV-1**	71	429	332	230	176	176	685	92	2166	80
**EKV-2**	69	427	217	229	175	168	630	125	2120	60
**BASV**	ND	429	215	218	216	171	629	94	2123	ND
**TIBV**	72	428	308	253	170	149	662	109	2119	170
**CPV**	67	425	274	244	173	161	658	103	2128	250
**BHV**	72	428	312	253	170	159	659	117	2116	168
**SWBV**	ND	428	292	248	170	162	662	105	2120	ND
**BAV**	ND	428	309	253	170	159	662	109	2119	ND

^1^ Values represent the number of amino acid residues, except for the 3′-trailer and 5′- leader columns, in which the number of nucleic acid residues (nt) are indicated. ND, not determined.
